# Supplementary material for: From sequence to enzyme mechanism using multi-label machine learning
Source: BMC Bioinformatics. 2014 May 19;15:150. doi: 10.1186/1471-2105-15-150 (PMC4229970; doi:10.1186/1471-2105-15-150)
Supplement: Additional file 2 — Java code of ml2db. Additional file ml2db_code.tar.gz contains the Java source code to run the multi-label machine learning experiments and save the results to database. The code’s Javadoc is included. [file 1471-2105-15-150-S2.zip › additional file 2/ml2db/ml2db/doc/main/ml2db/weka/package-tree.html]

main.ml2db.weka Class Hierarchy


JavaScript is disabled on your browser.


- Overview
- Package
- Class
- Use
- Tree
- Deprecated
- Index
- Help

- Prev
- Next

- Frames
- No Frames

- All Classes

# Hierarchy For Package main.ml2db.weka

Package Hierarchies:

- All Packages

## Class Hierarchy

- java.lang.Object
  - weka.core.AbstractInstance (implements weka.core.Instance, weka.core.RevisionHandler, java.io.Serializable)
    - weka.core.SparseInstance
      - main.ml2db.weka.IdentifiedSparseInstance
  - java.util.AbstractMap<K,V> (implements java.util.Map<K,V>)
    - java.util.TreeMap<K,V> (implements java.lang.Cloneable, java.util.NavigableMap<K,V>, java.io.Serializable)
      - uk.ac.standrews.utils.main.maputils.MapOfMaps
        - main.ml2db.weka.InstAttValueMap
  - main.ml2db.weka.AttributeFactory
  - main.ml2db.weka.DataSetChecker
  - main.ml2db.weka.DataSetDbLoader
  - main.ml2db.weka.DataSetGenerator
  - main.ml2db.weka.DataSetWriter
  - main.ml2db.weka.ImportArff
  - uk.ac.standrews.utils.main.Initialised (implements uk.ac.standrews.utils.main.Initialisable)
    - main.ml2db.weka.Arff
    - main.ml2db.weka.ArffProps
      - main.ml2db.weka.ArffPropsFromFile
  - main.ml2db.weka.InstancesFiller
  - main.ml2db.weka.JaccardDistanceStored (implements weka.core.DistanceFunction, java.io.Serializable)
  - main.ml2db.weka.JaccardDistanceTest
  - uk.ac.standrews.utils.main.database.Manager
    - main.ml2db.weka.DataSetManager
  - weka.core.NormalizableDistance (implements weka.core.DistanceFunction, weka.core.OptionHandler, weka.core.RevisionHandler, java.io.Serializable)
    - main.ml2db.weka.JaccardDistance (implements java.io.Serializable)
      - main.ml2db.weka.JaccardEuclideanDistance
  - uk.ac.standrews.utils.main.database.Table
    - main.ml2db.weka.ArffRecordsTable

- Overview
- Package
- Class
- Use
- Tree
- Deprecated
- Index
- Help

- Prev
- Next

- Frames
- No Frames

- All Classes
